# Structural and Mechanistic Bases of Viral Resistance to HIV-1 Capsid Inhibitor Lenacapavir

**DOI:** 10.1128/mbio.01804-22

**Published:** 2022-10-03

**Authors:** Stephanie M. Bester, Daniel Adu-Ampratwum, Arun S. Annamalai, Guochao Wei, Lorenzo Briganti, Bridget C. Murphy, Reed Haney, James R. Fuchs, Mamuka Kvaratskhelia

**Affiliations:** a Division of Infectious Diseases, Anschutz Medical Campus, University of Colorado School of Medicine, Aurora, Colorado, USA; b Division of Medicinal Chemistry and Pharmacognosy, College of Pharmacy, The Ohio State University, Columbus, Ohio, USA; University of Virginia; Rutgers-Robert Wood Johnson Medical School

**Keywords:** HIV-1, capsid, antiretroviral agents, drug resistance mechanisms, structure

## Abstract

Lenacapavir (LEN) is a long-acting, highly potent HIV-1 capsid (CA) inhibitor. The evolution of viral variants under the genetic pressure of LEN identified Q67H, N74D, and Q67H/N74D CA substitutions as the main resistance associated mutations (RAMs). Here, we determined high-resolution structures of CA hexamers containing these RAMs in the absence and presence of LEN. Our findings reveal that the Q67H change induces a conformational switch, which adversely affects the inhibitor binding. In the unliganded protein, the His67 side chain adopts the closed conformation by projecting into the inhibitor binding pocket and thereby creating steric hindrance with respect to LEN. Upon the inhibitor binding, the His67 side chain repositions to the open conformation that closely resembles the Gln67 side chain in the WT protein. We propose that the switch from the closed conformation to the open conformation, which is needed to accommodate LEN, accounts for the reduced inhibitor potency with respect to the Q67H CA variant. The N74D CA change results in the loss of a direct hydrogen bond and in induced electrostatic repulsions between CA and LEN. The double Q67H/N74D substitutions exhibited cumulative effects of respective single amino acid changes. An examination of LEN binding kinetics to CA hexamers revealed that Q67H and N74D CA changes adversely influenced the inhibitor binding affinity (*K_D_*) by primarily affecting the dissociation rate constant (*k_off_*). We used these structural and mechanistic findings to rationally modify LEN. The resulting analog exhibited increased potency against the Q67H/N74D viral variant. Thus, our studies provide a means for the development of second-generation inhibitors with enhanced barriers to resistance.

## INTRODUCTION

HIV-1 capsid protein (CA) is an attractive, yet clinically unexploited, therapeutic target. Extensive drug discovery efforts have resulted in the development of a potent, small molecule HIV-1 CA inhibitor, lenacapavir (LEN; GS-6207) (1, 2). In cell culture assays, LEN exhibits picomolar potency against all subtypes of HIV-1, including the strains resistant to current antiretroviral therapies. The interim results from ongoing phase 2/3 clinical trials have revealed that the subcutaneous administration of LEN with a 6-month dosing interval enables high rates of HIV-1 suppression in heavily treatment-experienced patients with multidrug resistant HIV-1 infection. These findings have highlighted LEN as a promising, long-acting agent that could complement current antiretroviral compounds to treat people living with HIV-1.

Mechanistic studies have revealed that LEN and its close analog GS-CA1 target the multifaceted roles of CA in HIV-1 biology (1–5). During virus ingress, these inhibitors impaired multiple CA dependent steps, including reverse transcription (EC_50_ value of 5 to 50 nM), nuclear import (EC_50_ value of 0.5 to 5 nM), and integration (EC_50_ value of 0.05 to 0.5 nM) (1, 2, 4). LEN and GS-CA1 rigidified intrahexamer and interhexamer interactions, which in turn compromised the functionally essential pliability of mature CA lattices (4, 6). During virion maturation, the inhibitors induced the accelerated, hyper-assembly of CA which resulted in improperly matured, inactive virions (1, 2).

High-resolution co-crystal structures of LEN in a complex with cross-linked CA hexamers revealed that the inhibitor binds in a hydrophobic pocket formed by two adjacent CA subunits (1, 4). LEN establishes extensive hydrophobic and electrostatic interactions with both CA subunits (referred to as CA1 and CA2) including two cation-π interactions with Arg173 and Lys70 and a hydrogen-bonding network with the side chains of Asn57, Lys70, and Asn74 of CA1 as well as Ser41, Gln179, and Asn183 of CA2 (Fig. S1). The pyridinium ring (R1) engages with the CA1 N-terminal domain (NTD) residues Asn53, Gly106, and Thr107, and the 2-(Methanesulfonyl)-2-methylpropane group of R1 engages with CA2-NTD residues Ile37 and Pro38 as well as CA1-NTD residue Thr54. The indazole ring (R2) interacts with several CA1-NTD residues, including Lys70, Ile73, Ala105, Thr107, and Tyr130. The difluorobenzyl group (R3) docks deep into the hydrophobic pocket of CA1-NTD, which is comprised of residues Leu56, Val59, Met66, Leu69, Lys70, Ile73, and Tyr130. The cyclopenta-pyrazole ring (R4) interacts with CA1-NTD and CA2 C-terminal domain (CTD) residues Gln63, Met66, Gln67, Lys70, Tyr169, Leu172, and Arg173.

10.1128/mbio.01804-22.1 FIG S1 LEN interactions with HIV-1 CA. (A) Chemical structure of LEN, highlighting the key interactions of LEN with wild-type (WT) CA hexamer residues through dashed lines, with the major ring systems labeled R1 to R4 (PDB ID: 6VKV). The key interacting residues from CA subunits 1 (CA1) and 2 (CA2) are indicated in grey and blue, respectively. Atom labels for LEN are shown in navy. (B) Gln67 and Asn74 (green) in the context of LEN (orange) bound to CA1 (light grey). Download FIG S1, TIF file, 2.9 MB. Copyright © 2022 Bester et al. 2022 Bester et al. https://creativecommons.org/licenses/by/4.0/ This content is distributed under the terms of the Creative Commons Attribution 4.0 International license.

Cell culture-based viral breakthrough assays have identified resistance associated mutations (RAMs) in close proximity of the LEN binding site on CA (1, 2). Q67H and N74D CA substitutions emerged as the main RAMs, whereas additional substitutions (L56I, M66I, Q67H/N74S, and Q67H/T107N) were detected at lower frequencies. With the exception of the Q67H change, all other RAMs substantially compromised HIV-1 replication (1, 2).

Q67H and N74D CA substitutions have also emerged in ongoing clinical trials with participants who have received LEN (1, 7–9). These changes conferred substantial (6-fold to >1,000-fold) resistance to LEN compared with wild-type HIV-1 (1, 4). Understanding the structural basis behind the viral resistance to LEN is crucial for the development of second-generation inhibitors.

Here, we have determined high-resolution structures of CA hexamers containing the main RAMs, which, together with biochemical and virology experiments, allowed us to elucidate the distinct underlying mechanisms behind the viral resistance to LEN. Furthermore, we employed these findings to develop an improved compound with enhanced activity against the viral Q67H/N74D CA phenotype. Thus, our structures provide a means for rationally developing improved, highly potent CA inhibitors with enhanced barriers to resistance.

## RESULTS

### RAMs reduce LEN binding affinity to CA hexamers.

**FIG 1:**
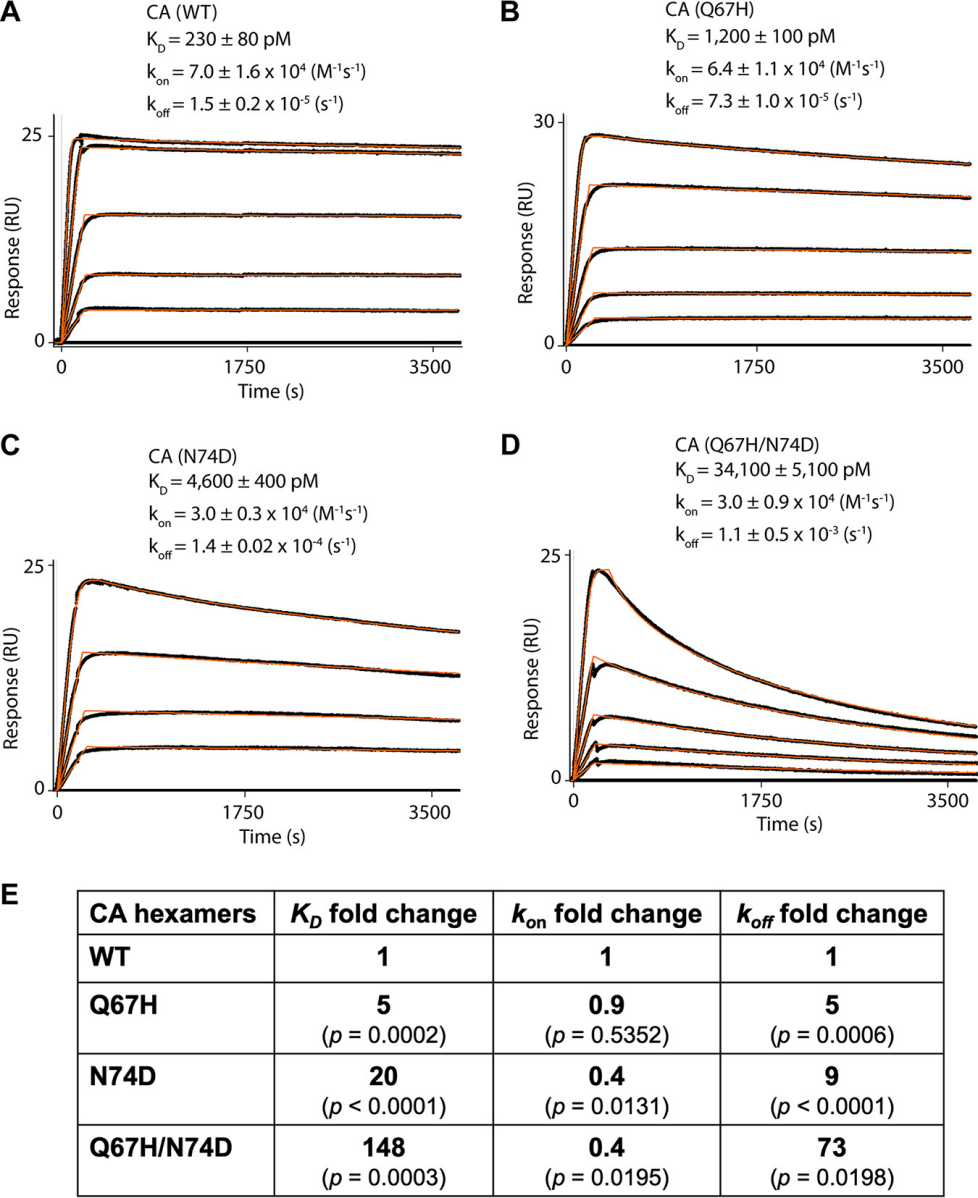
LEN binding to cross-linked HIV-1 CA hexamers. (A to D). Representative binding sensorgrams illustrating interactions between LEN with wild-type (WT) and mutant cross-linked HIV-1 CA hexamers evaluated by SPR detection. The binding data (black lines) were fit (orange lines) to a simple kinetic model with a mass transport added as needed. The mean ± standard deviation (SD) equilibrium dissociation constant (*K_D_*), association rate constant (*k_on_*), and dissociation rate constant (*k_off_*) values were determined from three independent experiments with comparable results. The LEN concentrations are 0.156, 0.312, 0.625, 1.25, and 2.5 μM in (A); 0.078, 0.156, 0.312, 0.625, and 1.25 μM in (B); 0.156, 0.312, 0.625, and 1.25 μM in (C); and 0.078, 0.156, 0.312, 0.625, and 2.5 μM in (D). (E) Fold change of the *K_D_*, *k_on_*, and *k_off_* values for the mutant CA variants compared to WT CA. *P* values indicate differences compared to CA(WT) hexamers.

To elucidate the mechanistic basis for resistance, we determined the equilibrium dissociation (*K_D_*), association rate (*k_on_*), and dissociation rate (*k_off_*) constants for LEN binding to cross-linked CA hexamers using surface plasmon resonance (SPR) (Fig. 1). The Q67H substitution resulted in an approximately 5-fold reduction in the *K_D_* value, due primarily to similarly reduced levels of *k_off_*, whereas the *k_on_* values for LEN association for Q67H and WT CA were similar (Fig. 1A and B). The N74D CA substitution adversely affected both the association (~2-fold) and dissociation (~10-fold) rate constants of LEN, resulting in an approximately 20-fold decrease in the inhibitor binding affinity to the CA(N74D) hexamer (Fig. 1C). The Q67H/N74D substitution exhibited even greater adverse effects on the LEN dissociation rate constants (*k_off_*) compared with those of the individual Q67H or N74D substitutions (Fig. 1D and E). Overall, LEN bound to Q67H/N74D CA displayed an approximately 150-fold reduced *K_D_* versus that of WT CA (Fig. 1E).

### Two distinct conformations of the His67 side chain regulate LEN binding.

**FIG 2:**
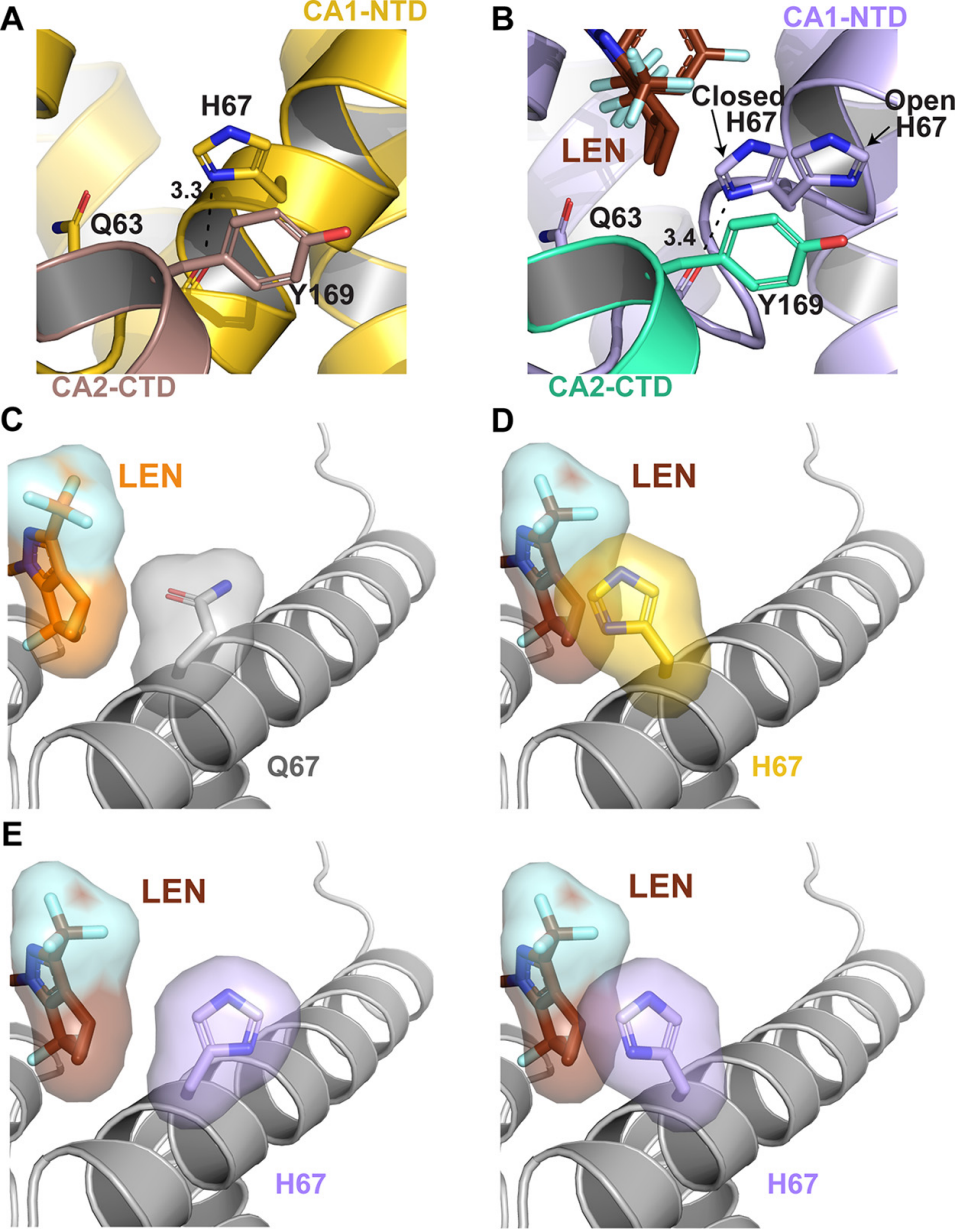
Structural basis for the LEN resistance of the CA(Q67H) hexamer. (A) X-ray crystal structure of the prestabilized CA(Q67H) hexamer (gold/dirty violet; PDB: 7RAR) with interactions between His67 and Tyr169 and Gln63 displayed. Hydrogen bonding interactions are denoted by black dashed lines, and distances are indicated. (B) X-ray crystal structure of prestabilized CA(Q67H) + LEN (lilac/seafoam + chocolate; PDB: 7RHN) displaying two conformations of His67, open and closed, and interactions between His67 and Tyr169 and Gln63. (C) Crystal structure of CA + LEN (light gray + orange; PDB: 6VKV) with surface renderings of LEN and Gln67 to demonstrate how they interact. (D) Overlay of CA(Q67H) (gold) and CA(Q67H) + LEN (not shown + chocolate) crystal structures with surface renderings of LEN and His67 to demonstrate how they would potentially interact. The darkened/shadowed surface rendering denotes an overlap in the space occupied by His67 and LEN. Cartoon regions are shown in light gray to highlight the substituted residue and LEN. (E) Crystal structure of CA(Q67H) + LEN (lilac + chocolate) with surface renderings of LEN and both conformations of His67 to demonstrate how they would potentially interact. The darkened/shadowed surface rendering denotes an overlap in the space occupied by His67 and LEN.

To examine the structural basis for the resistance of CA(Q67) to LEN, cross-linked CA hexamers containing the Q67H substitution were crystallized in the absence and presence of LEN (Fig. 2; Fig. S2; Tables S1 and S2). The intended mutation at residue 67 was present in both the apo 2.15 Å CA(Q67H) hexamer and in the 2.46 Å CA(Q67H) hexamer + LEN structures. Based on the 2F_o_-F_c_ density at 1 σ, the His67 side chain adopted two distinct conformations (Fig. 2A and B; Fig. S2). We will refer to the His67 conformations that point inside and outside the inhibitor-binding pocket as the closed and open conformations, respectively (Fig. 2B). In the absence of the inhibitor, we observed only the closed conformation (Fig. 2A), whereas the CA(Q67H) hexamer + LEN complex contained both the closed and open conformations of the His67 side chain (Fig. 2B). In the closed conformation, the imidazole ring of His67 forms π–π stacking interactions with Tyr169 and a hydrogen bond with the main chain carbonyl oxygen of Gln63. In contrast, the side chain of the open His67 conformation, which is shifted away from the inhibitor-binding pocket, does not appear to have any significant interactions (Fig. 2A and B). These observations explain why the closed conformation is preferred when the binding pocket is unoccupied. However, the closed His67 conformation sterically hinders LEN binding (Fig. 2C and D). In contrast, the open side chain conformation of His67 resembles the Gln67 side chain orientation in WT CA and, consequently, it is able to readily accommodate LEN. In addition to the presence of both conformations of His67 within the co-crystal structure of the CA(Q67H) hexamer + LEN, we also observed the incomplete density for the inhibitor, suggesting a partial occupancy of LEN within the binding pocket (Fig. S2). Collectively, these findings suggest that the His67 side chain needs to reposition from the closed to the open conformation to allow for the effective binding of the inhibitor.

10.1128/mbio.01804-22.2 FIG S2 Influence of the Q67H substitution on the LEN binding pocket of the CA hexamer. (A) X-ray crystal structure of the cross-linked CA(Q67H) hexamer (gold; PDB: 7RAR) with the 2mfo-dfc electron density scaled to 1σ (blue mesh) and the key residues of the binding pocket on CA displayed. (B) X-ray crystal structure of the cross-linked CA(Q67H) hexamer (lilac/seafoam) in complex with LEN (chocolate; PDB: 7RHN) with the 2mfo-dfc electron density scaled to 1σ (blue mesh). Hydrogen bonds are denoted by black dashed lines, and respective distances are indicated. (C and D) Surface rendering of the CA1-NTD for CA(WT) (PDB: 3H47) and CA(Q67H) (PDB: 7RAR), respectively, with the surface potential ranging from −5 to +5. Negatively charged regions are shown in red, and positively charged regions are shown in blue. Potentials were generated via the PDB2PQR server, and the surface was rendered using the adaptive Poisson-Boltzmann solver (APBS). Download FIG S2, TIF file, 2.2 MB. Copyright © 2022 Bester et al. 2022 Bester et al. https://creativecommons.org/licenses/by/4.0/ This content is distributed under the terms of the Creative Commons Attribution 4.0 International license.

10.1128/mbio.01804-22.9 TABLE S1 Summary of X-ray data collection and refinement statistics for apo cross-linked CA hexamers. Data were collected from a single crystal. Values in parentheses are from the highest-resolution shell. Download Table S1, DOCX file, 0.03 MB. Copyright © 2022 Bester et al. 2022 Bester et al. https://creativecommons.org/licenses/by/4.0/ This content is distributed under the terms of the Creative Commons Attribution 4.0 International license.

10.1128/mbio.01804-22.10 TABLE S2 Summary of X-ray data collection and refinement statistics for cross-linked CA hexamers in complex with inhibitors. Data were collected from a single crystal. Values in parentheses are from the highest-resolution shell. Download Table S2, DOCX file, 0.03 MB. Copyright © 2022 Bester et al. 2022 Bester et al. https://creativecommons.org/licenses/by/4.0/ This content is distributed under the terms of the Creative Commons Attribution 4.0 International license.

The Q67H substitution modestly enhances the positive electrostatic potential (Fig. S2). However, the imidazole ring in the open His67 conformation is substantially distanced from the inhibitor, and it does not form any electrostatic interactions with LEN. In the closed conformation, the imidazole ring decidedly creates steric hindrance with respect to LEN, whereas the contribution of the electrostatic potential change due to the Q67H substitution is less evident.

The Q67H change does not seem to affect other extensive electrostatic and hydrophobic interactions between LEN and the binding pocket of CA. Seven hydrogen bonds (the side chain oxygen of Ser41 to O40 of LEN, the side chain amine of Asn57 to O42 and N29 of LEN, the side chain oxygen of Asn57 to N3 of LEN, the side chain nitrogen of Lys70 to O3 and O57, and the side chain amine of Asn74 to O60 of LEN) and two cation-π interactions with Arg173 and Lys70 are fairly consistent between the two CA(Q67H) + LEN and CA(WT) + LEN (PDB 6VKV) structures (Fig. 2). We also note that a portion of αH9 and the loop preceding it are less well-defined in the CA(Q67H) + LEN structure compared with those of the CA(WT) + LEN structure.

### The N74D CA change introduces electrostatic repulsion to LEN binding.

**FIG 3:**
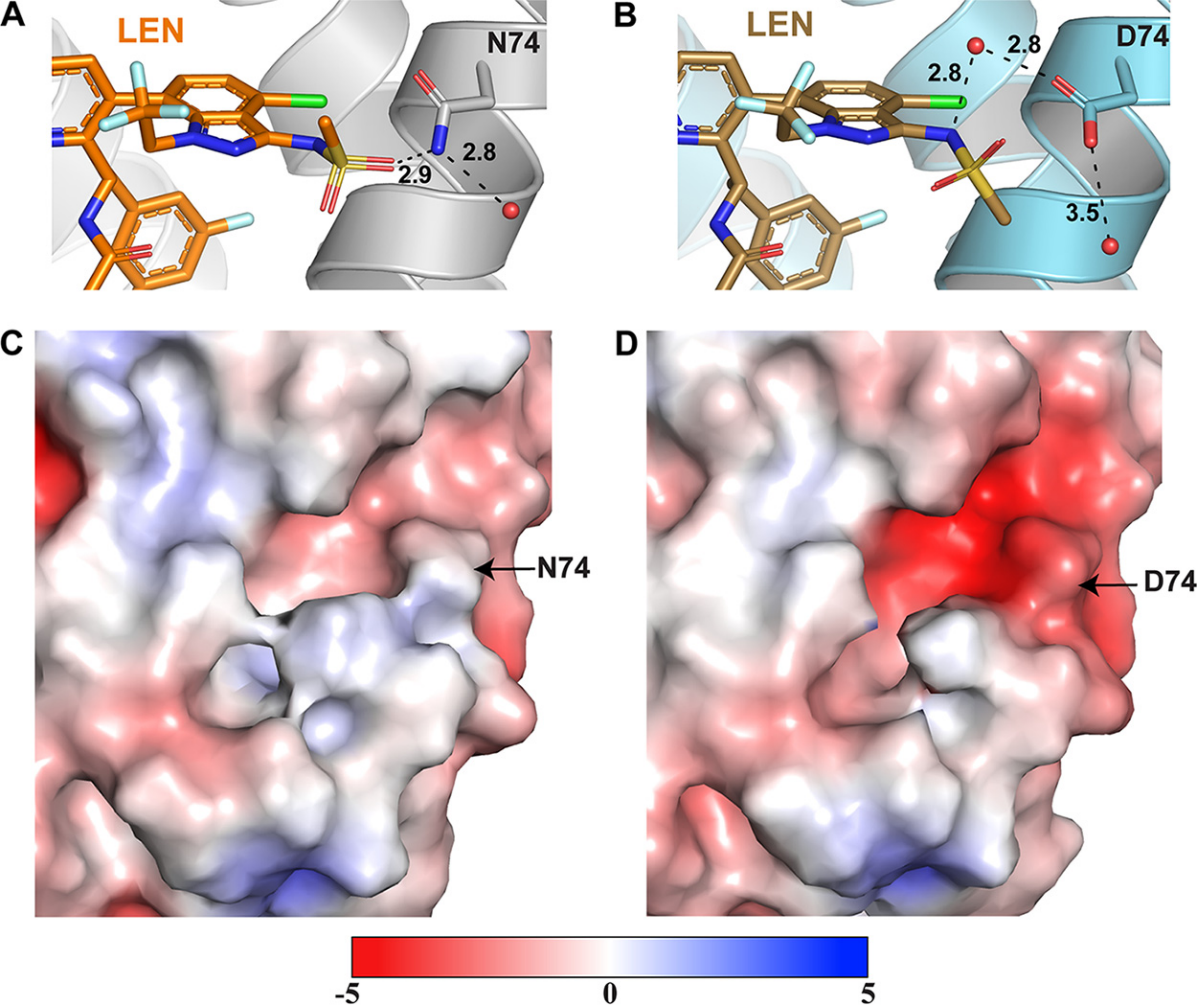
Structural basis for the LEN resistance of the CA(N74D) hexamer. (A) X-ray crystal structure of CA + LEN (light gray + orange; PDB: 6VKV) denoting the hydrogen bonding interaction between LEN and residue N74. Hydrogen bonding interactions are denoted by black dashed lines, and respective distances are indicated. (B) X-ray crystal structure of prestabilized CA(N74D) + LEN (steel blue + sand; PDB: 7RJ4) highlighting the hydrogen bonding interaction between LEN and residue Asp74 via a water molecule. (C and D) Surface rendering of the CA for CA(WT) (PDB: 3H47) and CA(N74D) (PDB: 7RMM), respectively, with the surface potential ranging from −5 to +5. Negatively charged regions are shown in red, and positively charged regions are shown in blue. Potentials were generated via the PDB2PQR server, and the surface was rendered using the adaptive Poisson-Boltzmann solver (APBS).

To elucidate the structural basis for how the N74D CA change confers resistance to LEN, we have determined the X-ray crystal structures of cross-linked CA(N74D) hexamers in the absence and presence of LEN at 1.97 and 3.32 Å resolutions, respectively (Fig. 3; Fig. S3; Tables S1 and S2). The overall secondary structures of CA(N74D) and CA(N74D) + LEN are highly similar to those of their WT counterparts. The N74D substitution primarily affects the positioning of the sulfonamide group (atoms 57 to 61) of LEN (Fig. 3A and B). The methyl group (C60) and oxygens (O57 and O59) are rotated with the oxygens further from residue 74 in CA(N74D) + LEN compared with CA(WT) + LEN.

10.1128/mbio.01804-22.3 FIG S3 Influence of the N74D substitution on the LEN binding pocket of the CA hexamer. (A) X-ray crystal structure of cross-linked CA(N74D) (pink; PDB: 7RMM) with the 2mfo-dfc electron density scaled to 1σ (blue mesh) and the key residues of the binding pocket on CA1-NTD displayed. (B) X-ray crystal structure of the cross-linked CA(WT) hexamer (steel blue/salmon) with N74D substitution in complex with LEN (sand; PDB: 7RJ4) with the 2mfo-dfc electron density scaled to 1σ (blue mesh). Hydrogen bonds are denoted by black dashed lines, and respective distances are indicated. Download FIG S3, TIF file, 2.4 MB. Copyright © 2022 Bester et al. 2022 Bester et al. https://creativecommons.org/licenses/by/4.0/ This content is distributed under the terms of the Creative Commons Attribution 4.0 International license.

There are two factors that are likely to influence the conformation of the sulfonamide group. First, in WT CA, is the side chain nitrogen of Asn74 hydrogen bonds with O60 of the sulfonamide group. This interaction is compromised by the Asn to Asp change, which allows the sulfonamide group to rotate. Asp74 can interact with the N61 of LEN via a water molecule, though these indirect inhibitor-protein contacts are likely to be substantially weaker than the direct hydrogen bonding between Asn74 and the sulfonamide group, as seen in the CA(WT) + LEN structure. Second, the N74D substitution results in a significantly more negative surface potential at this site, and its vicinity to the LEN binding site could adversely influence inhibitor binding (compare panels C and D in Fig. 3). Specifically, the LEN moiety, which is positioned near residue 74, is a negatively charged sulfonamide group. Therefore, the N74D change could create electrostatic repulsion with respect to the sulfonamide group of LEN. This electrostatic repulsion, as well as the loss of the direct hydrogen bonding interaction, are likely to be the primary causes of the resistance of CA(N74D) to LEN.

Despite these differences, CA(N74D) still forms many of the same interactions with LEN as seen with CA(WT), including several hydrogen bonding interactions with Ser41, Asn57, Lys70, Gln179, and Asn183 as well as two cation-π interactions with Arg173 and Lys70 (Fig. S3).

### The double Q67H/N74D substitutions reveal cumulative effects of respective single amino acid changes.

**FIG 4:**
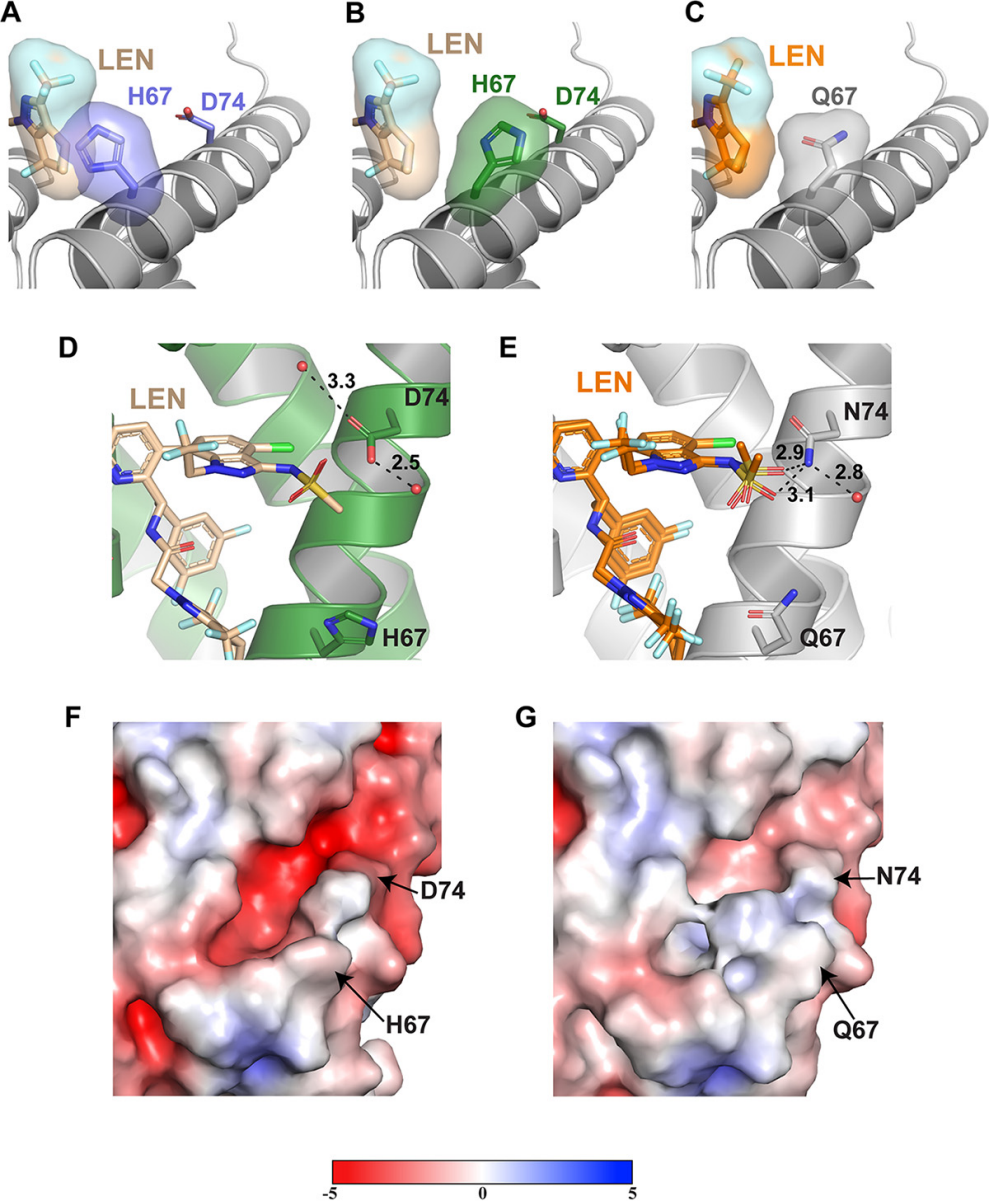
Structural basis for the LEN resistance of the CA(Q67H/N74D) hexamer. (A) X-ray crystal structure of the apo CA(Q67H/N74D) hexamer (slate purple; PDB: 7RHM) superimposed onto the CA(Q67H/N74D) + LEN (not shown + wheat) crystal structure with surface renderings of LEN and His67 to demonstrate how they would potentially interact. The darkened/shadowed surface rendering denotes an overlap in the space occupied by His67 and LEN. Cartoon regions are shown in light gray to highlight the substituted residue and LEN. (B) X-ray crystal structure of CA(Q67H/N74D) + LEN (forest green + wheat) with surface renderings of LEN and His67 to demonstrate their interaction. (C) Crystal structure of CA + LEN (light gray + orange; PDB: 6VKV) with surface renderings of LEN and Gln67 to demonstrate how they interact. (D) X-ray crystal structure of the prestabilized CA(Q67H/N74D) hexamer + LEN (forest green + wheat; PDB: 7RJ2), illustrating the lack of interactions between LEN and residue Asp74. (E) X-ray crystal structure of CA + LEN (light gray + orange; PDB: 6VKV) denoting the hydrogen bonding interaction between LEN and residue N74. Hydrogen bonding interactions are denoted by black dashed lines, and respective distances are indicated. (F and G) Surface renderings of the CA1-NTD for CA(Q67H/N74D) (PDB: 7RHM) and CA(WT) (PDB: 3H47), respectively, with the surface potential ranging from −5 to +5. Negatively charged regions are shown in red, and positively charged regions are shown in blue. Potentials were generated via the PDB2PQR server, and the surface was rendered using the adaptive Poisson-Boltzmann solver (APBS).

We have determined the X-ray crystal structures of cross-linked CA(Q67H/N74D) hexamers in the presence and absence of LEN at resolutions of 2.16 and 2.32 Å, respectively (Fig. 4; Fig. S4; Tables S1 and S2). Both of the changes introduced by the Q67H and N74D substitutions (Fig. 2 and 3) in the inhibitor-binding pocket were also evident in the CA(Q67H/N74D) structures (Fig. 4). In common with CA(Q67H) structures, two distinct conformations of His67 were observed in CA(Q67H/N74D) in the absence and presence of LEN (Fig. 4A and B). The unliganded CA(Q67H/N74D) exhibits the closed conformation of the His67 side chain, which is maintained through its hydrogen bonding with the carbonyl oxygen of the main chain of Gln63 and its π–π stacking interaction with Tyr169 (Fig. 4A; Fig. S4A). In contrast, His67 adopted an open conformation in CA(Q67H/N74D) + LEN (Fig. 4B; Fig. S4B). While we observed both the closed and the open conformations of the His67 side chain with partial occupancy of the inhibitor (Fig. S2B) with the CA(Q67H) + LEN structure, the CA(Q67H/N74D) + LEN structure revealed exclusively the open conformation of the His67 side chain and a strong density corresponding to the inhibitor (Fig. S4B), suggesting a greater occupancy of LEN than that observed in CA(Q67H) + LEN. These findings reinforce the notion that the switch from the closed to the open His67 conformation is needed to effectively accommodate LEN into the binding pocket.

10.1128/mbio.01804-22.4 FIG S4 Influence of the Q67H/N74D substitutions on the LEN binding pocket of the CA hexamer. (A) X-ray crystal structure of the cross-linked CA(Q67H/N74D) hexamer with substitution (slate purple/sage; PDB: 7RHM) with the 2mfo-dfc electron density scaled to 1σ (blue mesh) and the key residues of the binding pocket on CA displayed. (B) X-ray crystal structure of the cross-linked CA(Q67H/N74D) hexamer (forest green/light pink) in complex with LEN (wheat; PDB: 7RJ2) with the 2mfo-dfc electron density scaled to 1σ (blue mesh). Hydrogen bonds are denoted by black dashed lines and, respective distances are indicated. Download FIG S4, TIF file, 2.3 MB. Copyright © 2022 Bester et al. 2022 Bester et al. https://creativecommons.org/licenses/by/4.0/ This content is distributed under the terms of the Creative Commons Attribution 4.0 International license.

Similar to the single N74D change, the rotated conformation of the sulfonamide group was present in the CA(Q67H/N74D) + LEN structure (compare Fig. 4D with Fig. 4E). The oxygens (O57 and O60) are repositioned away from residue 74 in both CA(Q67H/N74D) and CA(N74D) compared with those observed in CA(WT). These changes are due likely to (i) the loss of hydrogen bonding interactions between the O60 of LEN and the amine of CA Asn74 and (ii) the enhanced negative surface potential introduced by the N74D change, which in turn could repulse the negatively charged sulfonamide group of LEN.

A comparative analysis of the single and double substitutions also revealed some relatively subtle differences. Asp74 interacts with Lys70 in unliganded CA(Q67H/N74D) but not in the CA(N74D) structure (Fig. S4). Unlike the CA(N74D) + LEN structure, in which the water molecule mediates interactions between the Asp74 and N61 of LEN, these interactions are absent in the CA(Q67H/N74D) + LEN structure, as the water molecule is too distant from Asp74 to form the hydrogen bond.

The double Q67H/N74D substitutions do not seem to largely influence the other extensive electrostatic and hydrophobic interactions of CA with LEN, as the five hydrogen bonds (with Ser41, Asn57, and Lys70) and two cation-π interactions (with Arg173 and Lys70) are quite similar to those of cross-linked CA(WT) + LEN (Fig. S4). In addition, unlike CA(WT) + LEN, a portion of αH9 and the loop preceding it are not well-defined in the CA(Q67H/N74D) + LEN structure. Therefore, the interactions between this CA region, which includes Q179 and N183, and LEN cannot be discerned in the context of CA(Q67H/N74D).

### Rational development of a LEN analog with enhanced activity against HIV-1_NL4-3_ containing the Q67H/N74D changes.

**FIG 5:**
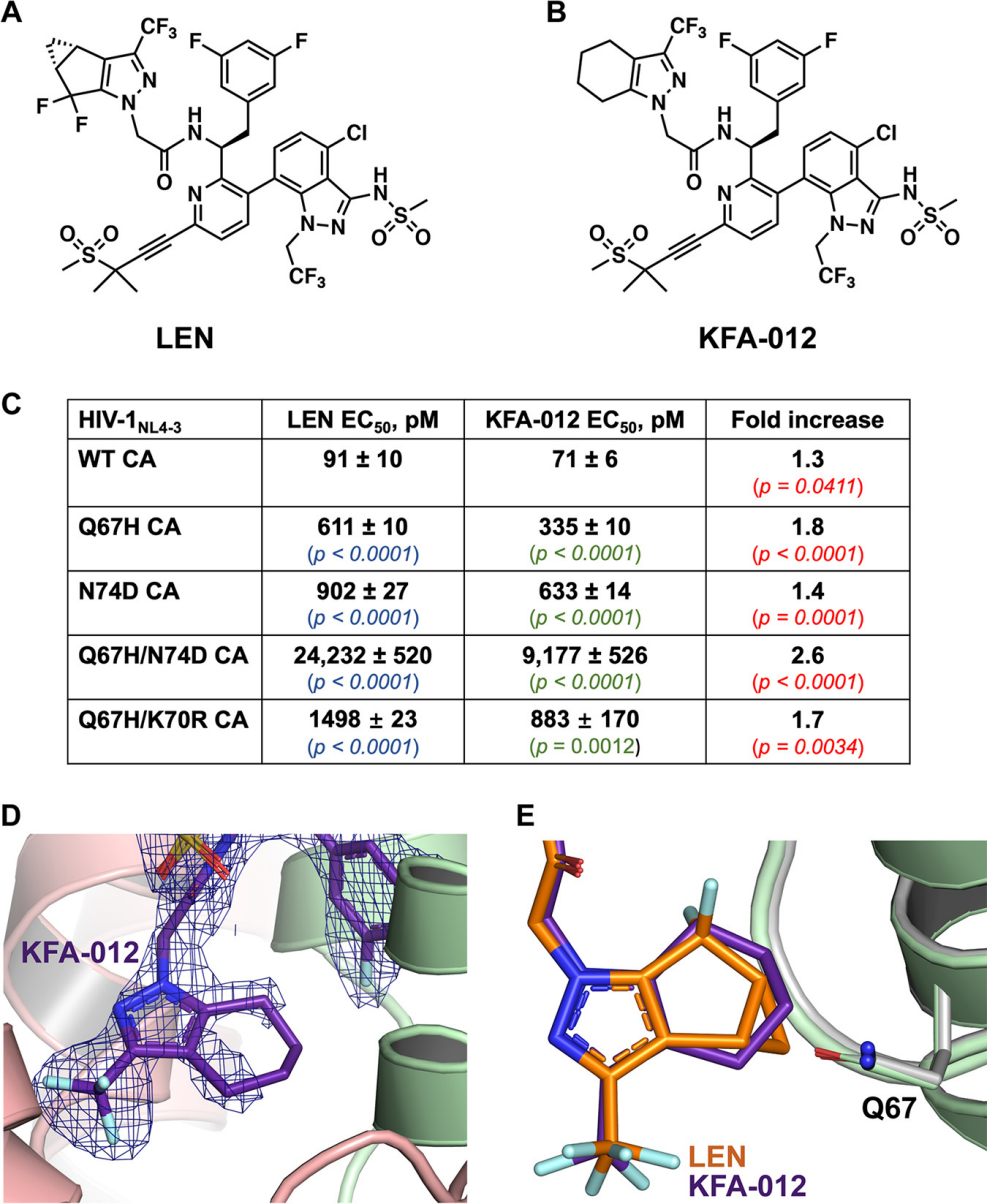
Comparison of the antiviral activities of LEN and KFA-012 against WT and CA mutants. (A) Chemical structure of LEN. (B) Chemical structure of the modified LEN-like compound KFA-012. (C) Early stage antiviral activities of LEN and KFA-012 were measured against pseudotyped HIV-1_NL4-3__RFP in MT-4 cells. *P* values in blue and green indicate differences compared to WT for LEN and KFA-012, respectively; *P* values in red show the statistical significance for the fold increases of the KFA-012 potency versus LEN potency with respect to the indicated CA variants. (D) X-ray crystal structure of the cross-linked CA(WT) hexamer (light green/peach) in complex with KFA-012 (dark purple) with the 2F_o_-F_c_ density scaled to 1σ (blue mesh; PDB: 7RMJ). (E) Superimposition of cross-linked CA(WT) hexamer in complex with KFA-012 (PDB: 7RMJ; light green + dark purple) and LEN (PDB 6VKV; gray + orange).

We attempted to exploit our structural findings to develop an improved compound with an enhanced barrier to resistance. The cyclopenta-pyrazole (R4) ring of LEN appears to be a good target for modification due to its close proximity to the Q67H substitution. Specifically, our structures indicate steric hindrance between the closed conformation of the His67 side chain and the cyclopropyl moiety of the cyclopenta-pyrazole ring. Therefore, we replaced the R4 ring (Fig. 5A) with a tetrahydroindazole ring, which also eliminated the cyclopropyl group and the two fluorine atoms present in LEN, resulting in KFA-012 (Fig. 5B; Fig. S5). This modification would allow for the R4 ring to be more flexible and, accordingly, less sensitive to the Q67H substitution. While both rings have 6 carbons attached to the pyrazole ring, the bridged system of the R4 ring of LEN is more rigid than is the cyclohexene ring of KFA-012 (Fig. 5A and B).

10.1128/mbio.01804-22.5 FIG S5 Synthesis of compound 12 (KFA-012). Synthetic schemes for the synthesis of modified subunit A (compound 3) as well as final compound 12 are shown (see Materials and Methods). Download FIG S5, DOCX file, 0.2 MB. Copyright © 2022 Bester et al. 2022 Bester et al. https://creativecommons.org/licenses/by/4.0/ This content is distributed under the terms of the Creative Commons Attribution 4.0 International license.

The antiviral activities of the resulting compound KFA-012 were examined against WT and LEN resistant viruses in MT-4 cells (Fig. 5C; Fig. S6). KFA-012 exhibited slightly better levels of activity against the WT HIV-1 and N74D CA substitutions than did LEN (Fig. 5C). The differences between the two compounds were relatively more pronounced with respect to the viruses containing the Q67H substitution. KFA-012 exhibited 1.8-fold and 2.6-fold higher potency toward HIV-1_(Q67H CA)_ and HIV-1_(Q67H/N74D CA)_ compared with the parental compound. Additionally, we have analyzed the early stage antiviral activities of KFA-012 against the Q67H/K70R and M66I CA variants, which emerged in clinical trials in patients receiving LEN (8). KFA-012 was ~1.7-fold more potent (EC_50_ ~883 pM) than LEN (EC_50_ ~1,498 pM) against HIV-1_(Q67H/K70R CA)_ (Fig. 5C), whereas the HIV-1_(M66I CA)_ virus was fully resistant to both KFA-012 and LEN (Fig. 6D and E). Next, we performed cytotoxicity assays with KFA-012 (Fig. S7). No cytotoxicity was observed with up to 781 nM KFA-012, whereas adverse effects on cell viability were detected with compound concentrations of >3 μM (Fig. S7). Collectively, these results (Fig. 5C; Fig. S7) indicate that the selectivity index of KFA-012 is >10,000.

10.1128/mbio.01804-22.6 FIG S6 Antiviral activities of LEN and KFA-012 against WT and drug-resistant CA variants. (A to C) Representative flow cytometry plots. MT4 cells were infected with pseudotyped HIV-1_RFP virus. An example of cell sorting plots is shown: (A) no infection (control); (B) infection in the presence of DMSO; and (C) infection in the presence of KFA-012. Gating was performed for live and single cells. The percentage of infected cells (RFP positive) was calculated as the ratio of P2 (PE positive population) to P1 (population of selected living cells). (D and E) Early stage antiviral activities of LEN (D) and KFA-012 (E) were measured against pseudotyped HIV-1_NL4-3__RFP viruses containing the indicated CA substitutions. The results are summarized in Fig. 5C. Download FIG S6, TIF file, 7.8 MB. Copyright © 2022 Bester et al. 2022 Bester et al. https://creativecommons.org/licenses/by/4.0/ This content is distributed under the terms of the Creative Commons Attribution 4.0 International license.

10.1128/mbio.01804-22.7 FIG S7 Cellular cytotoxicity of KFA-012. The indicated concentrations of KFA-012 were analyzed for their effects on the viability of MT4 cells using CellTiter-Glo (Promega, G7570). Control experiments with LEN are also shown. Download FIG S7, TIF file, 0.8 MB. Copyright © 2022 Bester et al. 2022 Bester et al. https://creativecommons.org/licenses/by/4.0/ This content is distributed under the terms of the Creative Commons Attribution 4.0 International license.

Consistent with the antiviral activity results (Fig. 5C), an SPR analysis of KFA-012 interactions with recombinant CA(WT) and CA(Q67H/N74D) hexamers revealed ~2-fold improved binding affinities of the compound compared those of with parental LEN (Fig. S8).

10.1128/mbio.01804-22.8 FIG S8 KFA-012 binding to cross-linked HIV-1 CA hexamers. (A and B) Representative binding sensorgrams illustrating the interactions between KFA-012 with the WT and Q67H/N74D mutant cross-linked HIV-1 CA hexamers, as evaluated by SPR detection. The binding data (black lines) were fit (orange lines) to a simple kinetic model with a mass transport component added as needed. The mean ± standard deviatino (SD) equilibrium dissociation constant (*K_D_*), association rate constant (*k_on_*), and dissociation rate constant (*k_off_*) values were determined from three independent experiments with comparable results. The KFA-012 concentrations were 0.031, 0.062, 0.125, 0.250, 0.5, 2, and 4 μM in (A) and were 0.062, 0.125, 0.250, 0.5, and 1 μM in (B). (C) Fold change of the *K_D_*, *k_on_* and *k_off_* values for the Q67H/N74D CA mutants compared to those of WT CA. *P* values indicate differences compared to the CA(WT) hexamers. Download FIG S8, TIF file, 1.9 MB. Copyright © 2022 Bester et al. 2022 Bester et al. https://creativecommons.org/licenses/by/4.0/ This content is distributed under the terms of the Creative Commons Attribution 4.0 International license.

Next, we have determined an X-ray crystal structure of the cross-linked CA(WT) hexamer in the presence of KFA-012 at a resolution of 2.27 Å (Fig. 5D; Table S2). When bound to the cross-linked CA(WT) hexamer, the modified ring of KFA-012 did not display complete 2F_o_-F_c_ density, which supports the flexibility of the cyclohexene ring (Fig. 5D). The elimination of the cyclopropyl group on the R4 ring appears to leave more space for the ring to maneuver within this site of the binding pocket (Fig. 5E). The increased flexibility afforded by the modified ring of KFA-012 could in turn account for its enhanced potency toward HIV-1_(Q67H CA)_ and HIV-1_(Q67H/N74D CA)_.

## DISCUSSION

Here, we report on the structural and mechanistic bases for how Q67H, N74D, and Q67H/N74D CA substitutions confer resistance to LEN. These CA substitutions have been previously identified as main RAMs following the serial passaging of HIV-1 in MT-2 cells with escalating LEN concentrations (1). Similar results were observed with a fixed concentration of LEN in human blood mononuclear cells (1). Furthermore, the Q67H and N74D CA substitutions have also emerged in clinical trials in some patents on LEN regimens (1, 7–9). Published studies (1, 4) have also shown that Q67H, N74D, and Q67H/N74D CA changes confer substantial resistance to LEN. However, the structural and mechanistic bases behind these observations were previously unknown.

### The Q67H resistance.

Our structural studies reveal that the side chain of His67 adopts two distinct closed and open conformations in the absence and presence of LEN, respectively. The closed conformation in the unliganded protein is stabilized by (i) π–π stacking interactions between the imidazole ring of His67 and Tyr169 and (ii) hydrogen bonding between His67 and the main chain carbonyl oxygen of Gln63. The closed His67 conformation creates a steric clash when superimposed with LEN. Therefore, upon LEN binding, the His67 side chain repositions to the open conformation, which closely resembles the Gln67 side chain in the WT protein. The need for a conformational switch from the closed to the open conformation to accommodate LEN could account for the reduced binding affinity (5-fold) and the similarly decreased potency (7-fold) of the inhibitor with respect to the Q67H CA variant.

The hydrophobic CA pocket, which houses LEN, is also the principal binding site for the FG motif containing host factors CPSF6, NUP153, and SEC24C. The superimposition of our apo CA(Q67H) structure with the FG-containing peptides from these three cellular proteins bound to cross-linked WT CA hexamers (10, 11) reveals the following. While the closed conformation of the His67 side chain extends into the binding pocket of the cellular factors, the imidazole group of His67 is sufficiently distanced from the CPSF6 (>4.1 Å) (PDB 4U0A), NUP153 (>5.6 Å) (PDB 4U0C), and SEC24C (>3.3 Å) (PDB 6PU1) peptides to avoid steric hindrance. These observations are consistent with antiviral assays, which indicate that the Q67H CA change does not detectably affect HIV-1 replication (1).

### The N74D resistance.

The N74D CA substitution has been extensively explored by virology and biochemistry assays for its critical role in interactions with cellular cofactors CPSF6 and SEC24C (10–13). Yet, the structure of the N74D mutant protein has not been reported previously. Here, we present X-ray crystal structures of CA hexamers containing the N74D substitution in the presence and absence of LEN, which reveal structural bases for both LEN resistance and deleterious effects of this CA substitution on CPSF6 and SEC24C binding.

Two contributing factors that render N74D CA resistant to LEN include: (i) the loss of direct hydrogen bonding between Asn74 in WT CA and O60 of LEN and (ii) electrostatic repulsions between the enhanced negative charge on the CA surface rendered by the N74D change and the sulfonamide group of LEN. As a result, the N74D substitution decreased LEN binding affinity to CA hexamers by ~20-fold and virus susceptibility to the inhibitor by ~10-fold.

Superimposition of our apo CA(N74D) structure with the FG-motif containing peptides of CPSF6 and SEC24C bound cross-linked WT CA hexamers helps us to better understand how the N74D change compromises CA binding to these cellular proteins (10). WT CA Asn74 forms three hydrogen bonds with each cofactor, including two interactions involving the side chain oxygen of Asn74 as well as one bond formed by the side chain amine of Asn74 and the main chain carbonyl oxygen of the Leu315 of CPSF6 (PDB 4U0A) or the Leu230 of SEC24C (PDB 6PU1). The two interactions formed by the side chain oxygen of Asn74 are not expected to be substantially affected by the substitution to Asp74. In contrast, the N74D change is expected to eliminate the bond with the Leu315 of CPSF6 (PDB 4U0A) and the Leu230 of SEC24C (PDB 6PU1). In addition, the CA substitution would introduce electrostatic repulsions between the side chain oxygen of Asp74 and the main chain carbonyl oxygen of the Leu315 of CPSF6 or the Leu230 of SEC24C. Accordingly, the N74D change impairs CA binding to both CPSF6 and SEC24C. In contrast, with these cofactors, the N74D substitution does not affect NUP153 binding to HIV-1 CA (10), as this CA residue is at least 7.5 Å away from the NUP153 peptide (PDB 4U0C).

### The Q67H/N74D resistance.

LEN dose escalation studies revealed an initial emergence of N74D followed by the Q67H/N74D variant at higher concentrations of the inhibitor (1). Our findings uncover structural and mechanistic bases for these observations. The CA(Q67H/N74D) and CA(Q67H/N74D) + LEN structures reveal combined effects seen with the two individual substitutions, in which a conformational switch with the Q67H change and the electrostatic repulsions due to the N74D substitution collectively make the inhibitor-binding pocket less amenable to LEN. Accordingly, the Q67H/N74D substitutions reduced LEN binding affinity to CA hexamers by ~150-fold compared to that of its WT counterpart, whereas individual N74D (~20-fold) and Q67H (~6-fold) changes exhibited lesser effects.

### The LEN analog KFA-012 exhibits enhanced potency against the drug-resistant variants.

Working toward a longer-term goal of developing improved CA inhibitors with higher barriers to resistance, we exploited our structural findings to modify LEN. Specifically, we changed the cyclopenta-pyrazole (R4) ring, which contains a rigid bridged ring system, to a more flexible tetrahydroindazole ring, which could potentially reduce the adverse effects of the Q67H conformational switch on the inhibitor binding. The high-resolution crystal structure of KFA-012 bound to the cross-linked CA(WT) hexamer revealed that the tetrahydroindazole ring, which, in contrast to LEN, lacks the rigid cyclopropyl group on the R4 ring and two fluorine atoms, has more space for maneuverability within the CA binding pocket than does LEN (Fig. 5D and E). In turn, the tetrahydroindazole ring of KFA-012 could be less affected by the Q67H substitution than the cyclopenta-pyrazole ring (R4) of LEN. Consistent with this notion, KFA-012 exhibited an approximately 2-fold improved binding affinity to the CA(Q67H/N74D) hexamer and a similarly enhanced potency with respect to HIV-1_(Q67H/N74D CA)_, compared with the parental compound. While these are relatively modest improvements, and while HIV-1_(Q67H/N74D CA)_ is still largely resistant to KFA-012, our findings provide a proof-of-concept for the rational development of second-generation CA inhibitors.

## MATERIALS AND METHODS

### Chemistry, general methods.

Unless stated otherwise, the reactions were monitored by thin-layer chromatography (TLC) on silica gel plates (60 F_254_), using *n*-hexanes and ethyl acetate (EtOAc) and visualized with UV light or *p*-anisaldehyde. Tetrahydrofuran (THF), dimethylformamide (DMF), ethanol (EtOH), methanol (MeOH), and all other reagents were used as received from chemical vendors. Column chromatography was performed on a Teledyne ISCO CombiFlash NextGen 300^+^ Instrument with a RediSep Flash Column, using hexanes and ethyl acetate as the mobile phase unless otherwise stated. The solvents for chromatography are listed as percentages (vol/vol). The ^1^H and ^13^C NMR spectra were recorded on a Bruker DPX 400 spectrometer in CDCl_3_ or DMSO-*d_6_* solutions operating at 400 MHz for ^1^HNMR and 100 MHz for ^13^CNMR unless otherwise stated. Chemical shifts were reported in ppm on the δ scale, relative to residual CHCl_3_ (δ = 7.26 for ^1^H NMR and δ = 77.2 for ^13^C NMR) as an internal reference. Spin multiplicities are given as s (singlet), d (doublet), t (triplet), and m (multiplet), as well as b (broad). Coupling constants (J) are given in hertz (Hz). High resolution mass spectra were measured on a Waters Synapt G2-Si HDMS. Trace analyses were obtained using a Shimadzu Prominence HPLC system. LEN was prepared in 12 steps (longest linear sequence) as previously described (4).

**Synthesis of 2-(3-(trifluoromethyl)-4,5,6,7-tetrahydro-1H-indazol-1-yl)acetic acid, compound 3.** See Fig. S5. A 1 M solution of lithium hexamethyldisilazide (LiHMDS) in THF (6.11 mL, 6.113 mmol) was cooled in an ice bath with stirring under argon. A solution of cyclohexanone, compound 1 (500.00 mg, 5.094 mmol), in anhydrous THF (12.70 mL) was then added dropwise via an addition funnel to the LiHMDS solution. The whole mixture was stirred for 30 min with cooling under argon at 0°C. Ethyl trifluoroacetate (0.66 mL, 5.604 mmol) was dropwise added to this mixture, and the mixture was stirred for a further 2 h at ambient temperature. After the complete consumption of the starting material (monitored by TLC), the mixture was partitioned between saturated aqueous NH_4_Cl solution (20.00 mL) and EtOAc (20.00 mL). The layers were separated, and the aqueous layer was extracted with EtOAc (10.00 mL × 2). The combined organic layers were dried over anhydrous Na_2_SO_4_, and the solvent was removed by rotary evaporation to give the diketone (not shown) as a brown oil, which was used directly in the next step.

The crude material from the previous step was dissolved with EtOH (10.20 mL) in a 100-mL flask. 4*N* HCl in 1,4-dioxane (2.55 mL, 10.188 mmol) was added to the solution, and this was followed by the addition of ethyl hydrazinoacetate hydrochloride (1.20 g, 7.641 mmol). The resulting mixture was heated at 70°C overnight. The EtOH was removed under reduced pressure, and the residue obtained was dissolved in EtOAc (50.00 mL). The whole mixture was transferred into a separatory funnel and washed with an aqueous saturated NaHCO_3_ solution (15.00 mL) and water (15.00 mL). The aqueous layers were combined and extracted with EtOAc (2 × 50.00 mL). The combined organic layer was dried with Na_2_SO_4_. After filtering, the solvent was removed by rotary evaporation to give the resulting ester, compound 2, as a yellow oil, which was used directly in the next step.

To a solution of the crude mixture from the previous step, 2*N* LiOH aq. (5.10 mL, 10.190 mmol) was added in a 2:1 mixture of THF/MeOH (17.00 mL). The resulting mixture was stirred for 1 h and then concentrated to remove most of the THF and MeOH. The obtained aqueous mixture was acidified with 1*N* HCl aq. to adjust the pH from 2 to 3, and it was then extracted with EtOAc (15.00 mL × 2). The organic phase obtained was dried over Na_2_SO_4_, filtered and concentrated *in vacuo* to provide the title compound, compound 3 (0.73 g, 58% 3 steps), as a yellow solid. ^1^H-NMR (400 MHz, CDCI_3_) δ ppm: 4.89 (s, 2H), 2.60 (t, *J *=* *6.0 Hz, 2H), 2.54 (t, *J *=* *6.1 Hz, 2H), 1.90 to 1.81 (m, 2H), 1.82 to 1.72 (m, 2H); ^13^C-NMR (100 MHz, CDCl_3_) δ: 170.4, 139.4 (q, *J *=* *36.5 Hz), 123.0, 120.3, 120.5, 115.8, 49.9, 22.1, 21.9, 21.0, 19.8; HDMS [M+H]^+^. (*m/z*) = calculated 249.0851, found 249.0854.

**Synthesis of compound 12 (KFA-012).** See Fig. S5. The synthesis and characterization data fro (S)-1-(3-(4-chloro-3-(*N*-(methylsulfonyl)methylsulfonamido)-1-(2,2,2-trifluoroethyl)-1*H*-indazol-7-yl)-6-(3-methyl-3-(methylsulfonyl)but-1-yn-1-yl)pyridin-2-yl)-2-(3,5-difluorophenyl)ethan-1-aminium triflate, compound 11, has been reported (4). Crude compound 11 (crude,~35.0 mg, 0.0391 mmol), 2-(3-(trifluoromethyl)-4,5,6,7-tetrahydro-1*H*-indazol-1-yl)acetic acid, compound 3 (10.70 mg, 0.0430 mmol), and diisopropylethylamine (20 μL, 0.117 mmol) were charged in a round bottom flask and dissolved in DMF (0.50 μL). Then, 1-hydroxybenzotriazole (15.60 mg, 0.041 mmol) was added. The reaction mixture was stirred at ambient temperature overnight. The reaction mixture was diluted with water (10.00 mL) and extracted with EtOAc (10.00 mL × 3). The organic layers were washed with brine, dried, and concentrated. The residue was dissolved in EtOH (0.20 mL), and 39.00 μL of 2*N* NaOH aq. was added. The resulting mixture was stirred at ambient temperature for 30 min, diluted with water (10.00 mL), and acidified with 1*N* HCl aq. After extraction with EtOAc, the organic layer was washed with water, brine, and dried over Na_2_SO_4._ The residue was then purified by silica gel chromatography (30% EtOAc in n-hexanes) to give 20 mg of the titled compound, compound 12. ^1^H NMR (400 MHz, CDCl_3_) δ: 7.53 (s, 1H), 7.53 (s, 1H), 7.09 (d, *J *=* *8.4 Hz, 1H), 7.08 (d, *J *=* *7.8, Hz, 1H), 6.62 (tt, *J *=* *9.0, 2.3 Hz, 1H), 6.37 (d, *J *=* *7.5, 1H), 6.16 to 6.09 (m, 2H), 4.74 (q, *J *=* *8.0 Hz, 1H), 4.61 (d, *J *=* *16.5 Hz, 1H), 4.54 (d, *J *=* *16.5 Hz, 1H), 4.38 to 4.27 (m, 1H), 3.88 to 3.76 (m, 1H), 3.39 (s, 3H), 3.18 (s, 3H), 2.98 to 2.90 (m, 1H), 2.85 to 2.79 (m, 3H), 2.62 to 2.49 (m, 3H), 2.46 to 2.36 (m, 1H), 1.86 (s, 6×H), 1.83 to 1.70 (m, 4H); ^13^C-NMR (100 MHz, CDCl_3_) δ: 165.3, 162.9 (dd, *J *=* *249.4, 12.6 Hz), 157.9, 143.2, 141.8, 140.2, 140.1, 139.9 (q, *J *=* *20.3 Hz), 139.7, 138.8, 130.9, 130.4, 127.0, 126.8, 123.1, 121.6, 118.6, 116.0, 114.8, 112.3 (d, *J *=* *7.3 Hz), 112.0 (d, *J *=* *7.3 Hz), 102.4 (t, *J *=* *25.2 Hz), 88.8, 84.7, 58.0, 52.1, 52.0, 51.8, 50.8, 50.5, 41.6, 41.5, 38.6, 35.3, 29.7, 22.7, 22.6, 22.1, 21.8, 21.1, 19.8; HDMS [M+H]^+^. (*m/z*) = calculated 934.1858, found: 934.1866.

### Cells.

HEK293T (ATCC) were cultured in Dulbecco’s modified eagle medium (DMEM, Gibco) supplemented with 10% fetal bovine serum (FBS, Sigma–Aldrich) and 1% penicillin–streptomycin (PS, Gibco). MT4 (ATCC) cells were cultured in the Roswell Park Memorial Institute (RPMI) 1640 medium (Gibco) supplemented with 10% FBS and 1% PS. Cells were maintained in an incubator at 37°C and 5% CO_2_. All of the cell lines used in the study were tested monthly for *Mycoplasma* contamination.

### Plasmids and viral vectors.

For the antiviral assays, the HIV-1 based vector (pNL4-3_RFP) was used (12). The Q67H, N74D, Q67H/N74D, Q67H/K70R, and M66I CA changes were introduced in pNL4-3_RFP via site directed mutagenesis using a Quickchange II XL site-directed mutagenesis kit (Agilent Technologies) as per the manufacturer’s instructions.

### Virus production and antiviral assays.

For the virus stock production, we used previously described protocols (11). Briefly, HEK293T cells (seeded at concentrations of 3 to 5 × 10^5^ cells/mL) were cotransfected with plasmids pNL4-3_RFP and pMD.G using the X-treme Gene HP (Roche) reagent as per the manufacturer’s instructions. The medium was removed and replaced with fresh medium at 12 to 16 h posttransfection and incubated at 37°C. The virus containing supernatant was harvested at 72 h posttransfection, clarified, filtered through a 0.45 μm filter, concentrated using Amicon Ultra Centrifugal Filters (Sigma), and stored at −80°C until further use.

The antiviral activities of LEN and KFA-012 against HIV-1 viruses were evaluated in MT-4 cells. MT-4 cells were seeded (5 × 10^4^ cells/well) in a 24-well plate and were 2-fold serially diluted via inhibitor concentrations or DMSO added to the wells. Subsequently, the cells were infected with VSV-G pseudotyped HIV-1 and incubated at 37°C for 3 to 4 h. Then, the cells were washed by centrifugation, resuspended with fresh medium containing 2-fold serially diluted inhibitor concentrations or DMSO, and incubated at 37°C for 48 h. Finally, the cells were collected and washed with PBS (10% FBS, 1 × PS) and fixed with 2% paraformaldehyde in PBS. The infectivity was measured by flow cytometry and analyzed using BD FACSDiva software (v.8.0.1). Effective inhibitor concentration as 50% inhibition (EC_50_) values were determined using the Origin 2019 (v9.6) software package. All virus infections were done in the presence of 8 μg/mL Polybrene, and the values were expressed as mean ± standard deviation (SD).

### Cytotoxicity assay.

MT4 cells in complete RPMI were mixed with the indicated concentrations of compounds on 96-well plates (1 × 10^4^ cells per well). Cells were kept in a humidified 37°C incubator for 2 days. The effect of test compounds on cell viability was measured using CellTiter-Glo (Promega, G7570).

### Expression, purification and assembly of recombinant CA.

RAMs were engineered in the background of CA(A14C/E45C/W184A/M185A), which allowed us to prepare cross-linked CA hexamers for X-ray crystallography and SPR experiments (14, 15). CA(A14C/E45C/W184A/M185A) is referred here to as CA(WT). Q67H, N74D, and Q67H/N74D changes were introduced in pET3a using a QuikChange XL Site-directed mutagenesis kit (Aligent). CA(WT) and the mutant proteins were expressed from pET3a in BL21-DE3 cells and purified, as described previously (14, 15), via two column chromatography using HiTrap SP-Sepharose High Performance and HiTrap Q-Sepharose High Performance 5 mL columns (GE Healthcare) for hexamer formation. The intersubunit disulfide-stabilized WT and mutant CA hexamers were assembled as previously published (15). The assembled hexamers were purified further through size exclusion chromatography using a GE Healthcare HiLoad 16/600 Superdex 200 pg column with a buffer containing 20 mM Tris-HCl (pH 8.0) and 150 mM NaCl. WT and mutant CA hexamers were detected using nonreducing SDS-PAGE and were concentrated to ~10 to 12 mg/mL with a 50 kDa cutoff Amicon Ultra-15 Centrifugal concentrator for utilization in apo crystallization and surface plasmon resonance binding experiments, respectively. After size exclusion chromatography, CA hexamers were diluted with the aforementioned buffer to reach 0.167 μM. Then, LEN was added over time to reach a final concentration of 1 μM and a ratio of six compounds per hexamer. The complex was then concentrated to 10 to 12 mg/mL of hexamer with a 50 kDa molecular weight cutoff Amicon Ultra-15 Centrifugal concentrator for crystallization.

### Surface plasmon resonance.

Surface plasmon resonance biosensor binding experiments were performed using the Reichert 4-SPR. A nitrilotriacetic acid (NTA) sensor chip was conditioned with 40 mM NiSO_4_ at a flow rate of 25 μL/min for 3 min. Cross-linked 6×-His-CA hexamers (WT and mutant variants: Q67H, N74D, and Q67H/N74D) were immobilized on the NTA sensor chip via their C-terminal His-tags. The running buffer contained 0.01 M HEPES (pH 7.4), 0.15 M NaCl, 0.05% vol/vol Surfactant P20, and 5% DMSO. LEN was prepared by serially diluting the compound in 100% DMSO and then by adding running buffer (without DMSO) to reach a final DMSO concentration of 5% and the indicated concentrations of LEN and KFA-012. The sensor chip was regenerated with 350 mM EDTA and 50 mM NaOH. For each interaction, background binding and drift were subtracted via a NTA reference surface. Data were analyzed using Scrubber 2.0 and fit with a simple kinetic model with a term for mass transport.

### Statistical analysis.

The two-tailed *P* values were generated via unpaired *t* tests from mean, SD and N values using the GraphPad Calculator.

### X-ray crystallography.

Apo crystals of mutant variants (Q67H, N74D, and Q67H/N74D) of CA hexamer, co-crystals of mutant variants (Q67H, N74D, and Q67H/N74D) of CA hexamer with LEN, and co-crystals of the CA(WT) hexamer with KFA-012 were grown via hanging drop vapor diffusion at 4°C with an equal volume of crystallization buffer. The crystallization buffers contained 1.5 to 12% PEG3350, 0.050 to 0.425 M NaI, 0.1 M sodium cacodylate (pH 6.5), and 6% glycerol. The crystals were hexagonal rods and appeared within 2 weeks. The cryogenic solutions for the crystals consisted of crystallization buffer with additional PEG3350 and glycerol to reach either 12 and 24%, respectively, or 20 and 10%, respectively. Crystals were flash cooled in liquid nitrogen. The data were collected at the Advanced Light Source, Beamline 4.2.2 (Macromolecular Crystallography; MBC) at 100 K and at a wavelength of 1.00003 Å. The data were processed and scaled using XDS (16). PHASER (17) from the PHENIX suite (18) was used to perform molecular replacement, using PDB 6VKV as a search model. The structures were refined using repeated cycles of model building and refinement via COOT (19) and phenix.refine (18), respectively. TLS Motion Determination (TLSMD) was utilized to analyze the flexibility of the structures. This analysis provided the TLS parameters in phenix.refine that assisted in refining the structures for the anisotropic displacements (18). Ligands were individually oriented into the structures based on the F_o_-F_c_ omit map density at 3 σ and were then refined with phenix.refine to ensure that they fit the 2F_o_-F_c_ density at 1 σ (18). Water molecules were originally added through the Find Water COOT program, but each water was then evaluated individually to ensure that it fit the 2F_o_-F_c_ density at 1*σ* (20). Molprobity was used to assess the final model of each structure and ensure its quality (21). The coordinates are deposited in the Protein Data Bank under accession codes 7RAR, 7RMM, 7RHM, 7RHN, 7RJ4, 7RJ2, and 7RMJ. The data collection and refinement statistics are given in Tables S1 and S2.

### Data availability.

All data are available in the manuscript or in the supplemental material. The crystal structures are deposited into the PDB under the accession numbers 7RAR, 7RMM, 7RHM, 7RHN, 7RJ4, 7RJ2, and 7RMJ.
