# Supplementary figures and images for: Structural and Mechanistic Bases of Viral Resistance to HIV-1 Capsid Inhibitor Lenacapavir
Source: mBio. 2022 Oct 3;13(5):e01804-22. doi: 10.1128/mbio.01804-22 (PMC9600929; doi:10.1128/mbio.01804-22)

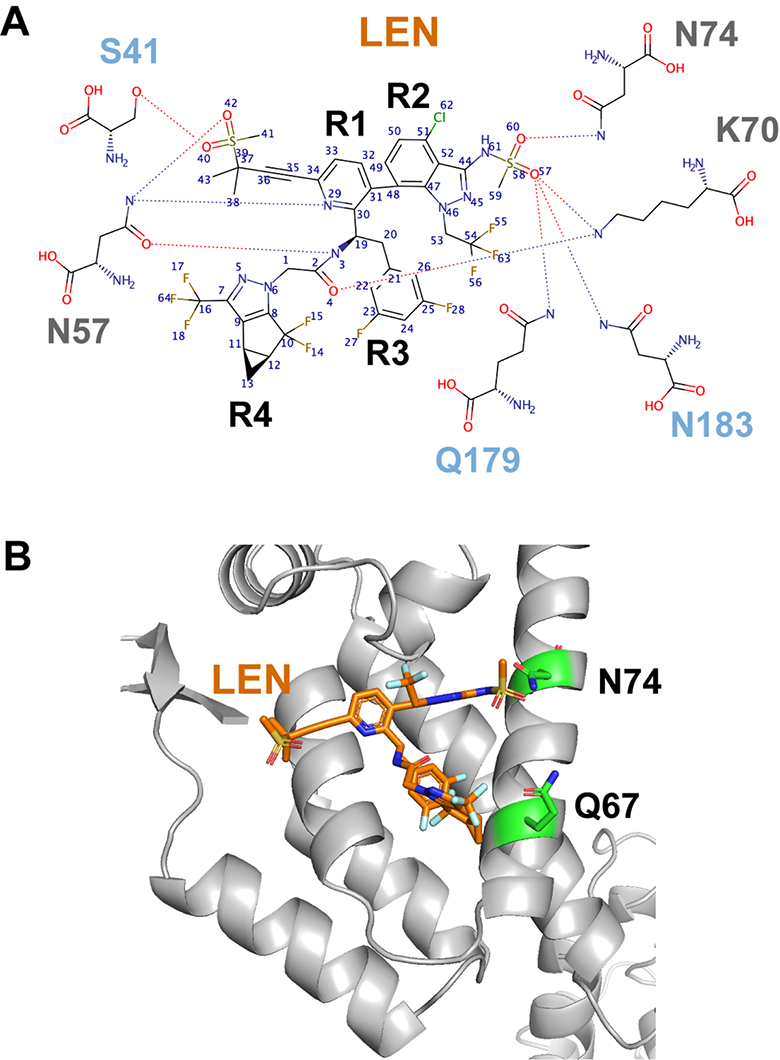

Supplement: FIG S1 [file mbio.01804-22-s0001.tif]

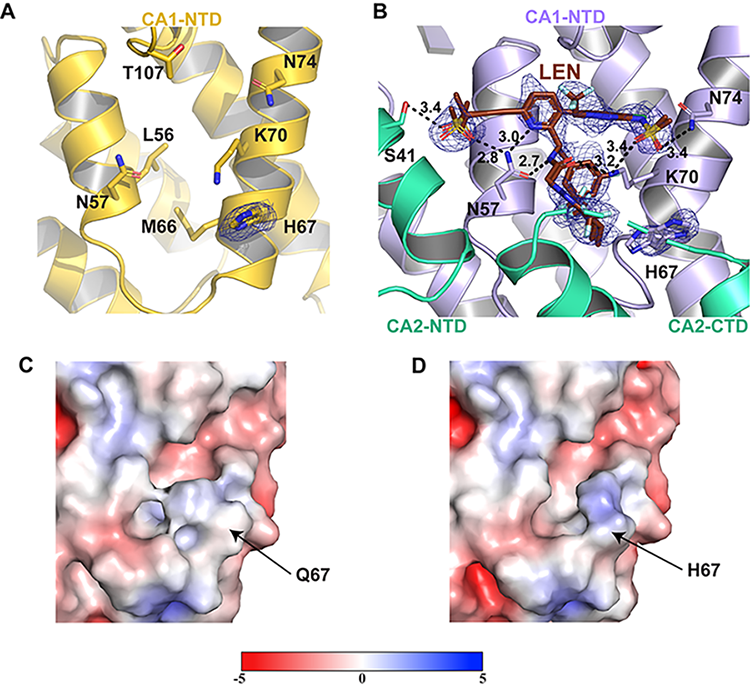

Supplement: FIG S2 [file mbio.01804-22-s0002.tif]

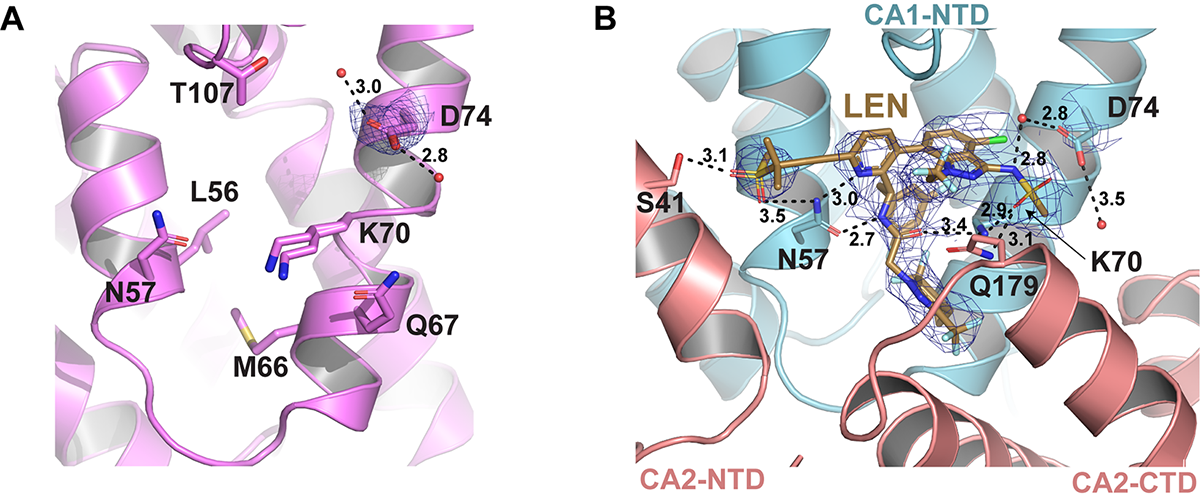

Supplement: FIG S3 [file mbio.01804-22-s0003.tif]

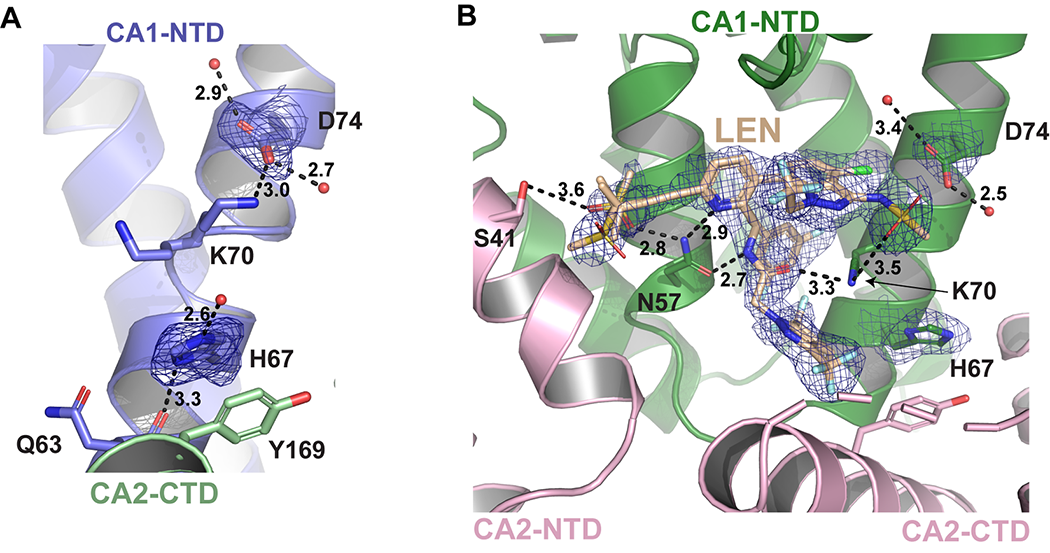

Supplement: FIG S4 [file mbio.01804-22-s0004.tif]

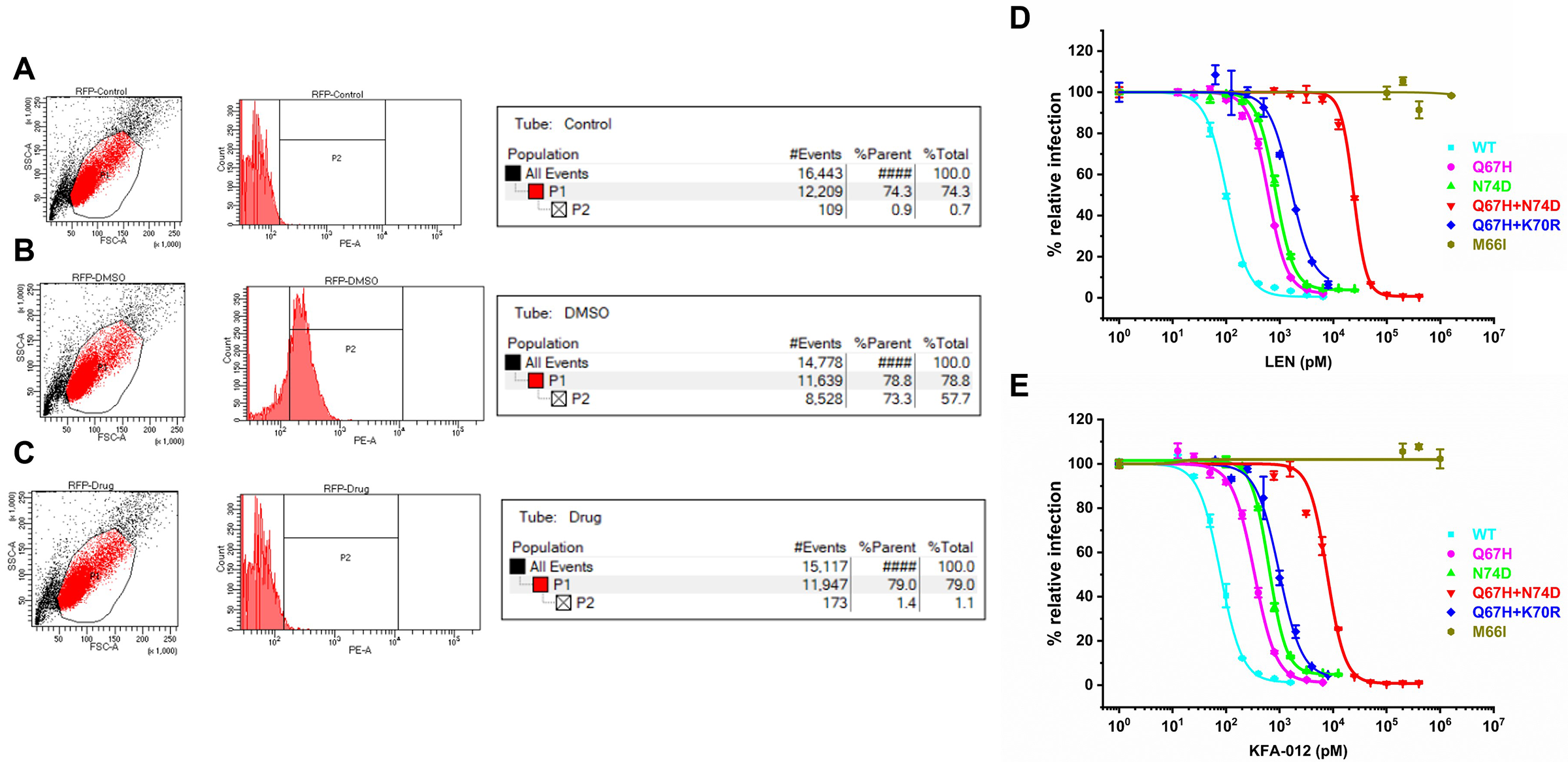

Supplement: FIG S6 [file mbio.01804-22-s0006.tif]

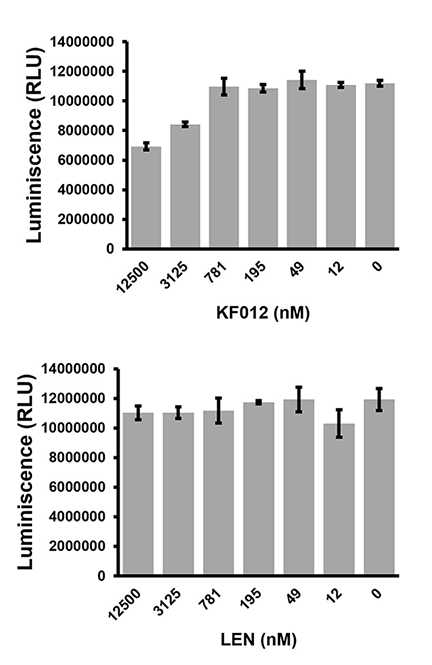

Supplement: FIG S7 [file mbio.01804-22-s0007.tif]

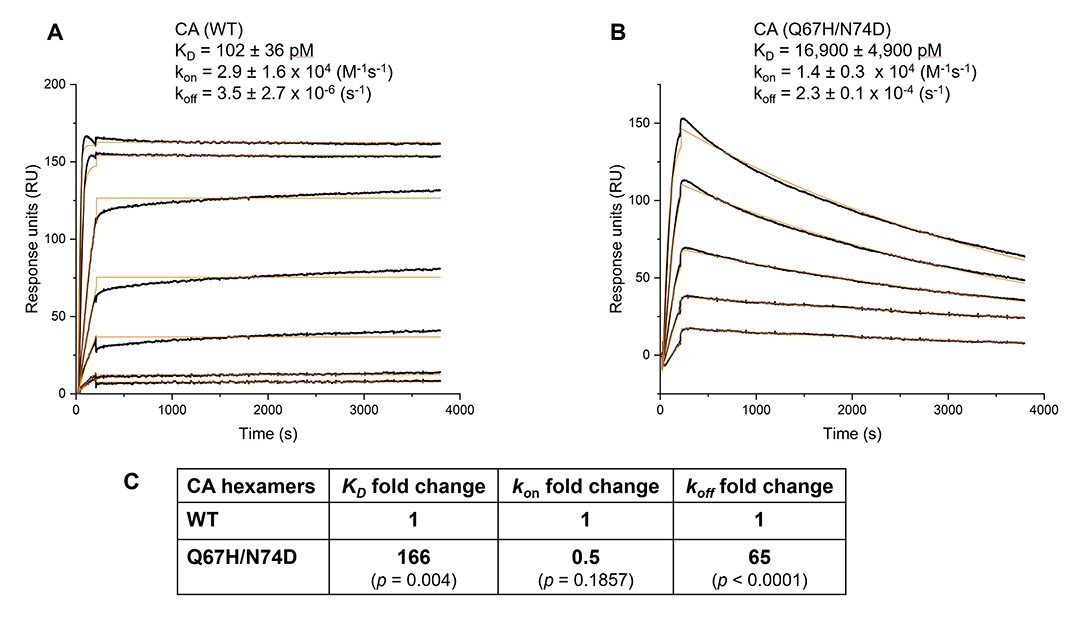

Supplement: FIG S8 [file mbio.01804-22-s0008.tif]
