# Supplementary material for: Structural and Mechanistic Bases of Viral Resistance to HIV-1 Capsid Inhibitor Lenacapavir
Source: mBio. 2022 Oct 3;13(5):e01804-22. doi: 10.1128/mbio.01804-22 (PMC9600929; doi:10.1128/mbio.01804-22)
Supplement: TABLE S1 [file mbio.01804-22-s0009.docx]

|  | CA(Q67H)  PDB ID: 7RAR | CA(N74D)  PDB ID:  7RMM | CA(Q67H/N74D)  PDB ID: 7RHM |
| --- | --- | --- | --- |
| **Data collection**  X-ray Source  Software  Wavelength | ALS 4.2.2  XDS  1.00003 Å | ALS 4.2.2  XDS  1.0000 Å | ALS 4.2.2  XDS  1.0000 Å |
| Space group | P6 | P6 | P6 |
| Unit cell dimensions |  |  |  |
| *a*, *b*, *c* (Å) | 91.85, 91.85, 57.78 | 157.36, 157.36, 56.58 | 92.12, 92.12, 57.84 |
| α, β, γ (°) | 90, 90, 120 | 90, 90, 120 | 90, 90, 120 |
| Resolution (Å)  No. total reflections  No. unique reflections | 46.75 – 2.15 (2.22-2.15)  165,261 (13,464)  15,259 (1,325) | 45.43 – 1.97 (2.02-1.97)  620,065 (40,916)  56,827 (4,002) | 46.83 – 2.16 (2.23-2.16)  164,090 (14,039)  15,160 (1,303) |
| R*_merge_*  CC1/2 | 0.182 (1.590)  0.997 (0.667) | 0.106 (1.308)  0.999 (0.639) | 0.219 (1.911)  0.995 (0.608) |
| *I*/ σ*I* | 12.2 (1.5) | 15.8 (1.6) | 10.8 (1.3) |
| Completeness (%) | 100 (100) | 100 (100) | 100 (100) |
| Multiplicity | 10.8 (10.2) | 10.9 (10.2) | 10.8 (10.8) |
|  |  |  |  |
| **Refinement** |  |  |  |
| Resolution (Å) | 46.75 – 2.15 (2.23–2.15) | 45.43 – 1.97 (2.04–1.97) | 46.83 – 2.16 (2.24–2.16) |
| No. reflections used in refinement  No. reflections used for R_free_ | 15,247 (1,511)  734 (71) | 56,783 (5,627)  2,788 (299) | 15,143 (1,505)  726 (65) |
| *R*_work_(%)  *R*_free_(%) | 20.80 (26.41)  23.98 (31.55) | 21.78 (29.10)  24.68 (33.85) | 23.13 (31.61)  25.96 (38.48) |
| No. non-hydrogen atoms | 1835 | 5243 | 1868 |
| Protein | 1649 | 4858 | 1686 |
| Ligand/ion | 10 | 23 | 9 |
| Water | 176 | 362 | 173 |
| Wilson B-factor  Average B-factors | 27.73  35.65 | 29.92  39.32 | 24.85  32.81 |
| Protein | 35.32 | 39.48 | 32.68 |
| Ligands/ions | 51.15 | 44.71 | 34.07 |
| Waters | 37.84 | 36.81 | 34.06 |
| R.m.s. deviations |  |  |  |
| Bond lengths (Å) | 0.005 | 0.006 | 0.002 |
| Bond angles (°)  Ramachandran      Favored (%)      Allowed (%)      Outliers (%)  Rotamer outliers (%)  Clashscore | 0.72    98.54  1.46  0  0  5.44 | 0.83    99.02  0.98  0  0.19  3.09 | 0.44    96.73  3.27  0  0  3.57 |
