# Supplementary material for: Structural and Mechanistic Bases of Viral Resistance to HIV-1 Capsid Inhibitor Lenacapavir
Source: mBio. 2022 Oct 3;13(5):e01804-22. doi: 10.1128/mbio.01804-22 (PMC9600929; doi:10.1128/mbio.01804-22)
Supplement: TABLE S2 [file mbio.01804-22-s0010.docx]

|  | CA(Q67H) + LEN  PDB ID: 7RHN | CA(N74D) + LEN  PDB ID: 7RJ4 | CA(Q67H/N74D) + LEN  PDB ID: 7RJ2 | CA(WT) + KFA-012  PDB ID: 7RMJ |
| --- | --- | --- | --- | --- |
| **Data collection**  X-ray Source  Software  Wavelength | ALS 4.2.2  XDS  1.00003 Å | ALS 4.2.2  XDS  1.00003 Å | ALS 4.2.2  XDS  1.0000 Å | ALS 4.2.2  XDS  1.0000 Å |
| Space group | P6 | P6 | P6 | P6 |
| Unit cell dimensions |  |  |  |  |
| *a*, *b*, *c* (Å) | 89.73, 89.73, 56.09 | 159.09, 159.09, 56.98 | 92.18, 92.18, 57.97 | 160.02, 160.02, 57.60 |
| α, β, γ (°) | 90, 90, 120 | 90, 90, 120 | 90, 90, 120 | 90, 90, 120 |
| Resolution (Å)  No. total reflections  No. unique reflections | 45.48 – 2.46 (2.56-2.46)  106,572 (12,081)  9,492 (1,063) | 46.32 – 3.32 (3.59-3.32)  108,913 (22,550)  12,427 (2,530) | 46.90 – 2.32 (2.40-2.32)  24,451 (2,373)  12,256 (1,189) | 50 – 2.27 (2.31-2.27)  39,080 (1,866) |
| R*_merge_*  CC1/2 | 0.200 (1.437)  0.997 (0.751) | 0.746 (3.739)  0.963 (0.838) | 0.048 (0.265)  0.998 (0.835) | 0.201 (0.777)  (0.601) |
| *I*/ σ*I* | 12.6 (1.6) | 3.1 (0.7) | 16.5 (3.0) | 9.8 (1.1) |
| Completeness (%) | 100 (100) | 99.9 (100) | 99.8 (99.9) | 99.7 (95.4) |
| Multiplicity | 11.2 (11.4) | 8.8 (8.9) | 2.0 (2.0) | 10.1 (3.4) |
|  |  |  |  |  |
| **Refinement** |  |  |  |  |
| Resolution (Å) | 44.87 – 2.46 (2.55–2.46) | 43.91 – 3.32 (3.44-3.32) | 39.92 – 2.32 (2.40–2.32) | 40.01 – 2.27 (2.35–2.27) |
| No. reflections used in refinement  No. reflections used for R_free_ | 9,482 (950)  458 (46) | 11,985 (842)  584 (49) | 12,251 (1,189)  604 (34) | 39,057 (3,781)  1,892 (187) |
| *R*_work_(%)  *R*_free_(%) | 24.17 (31.75)  28.84 (34.77) | 27.76 (36.11)  32.51 (35.50) | 19.95 (24.61)  24.51 (25.71) | 21.59 (27.87)  24.91 (31.84) |
| No. non-hydrogen atoms | 1802 | 5434 | 1971 | 5698 |
| Protein | 1620 | 5089 | 1649 | 5084 |
| Ligand/ion | 135 | 213 | 133 | 394 |
| Water | 47 | 132 | 189 | 220 |
| Wilson B-factor  Average B-factors | 35.29  44.38 | 82.93  84.54 | 28.84  36.58 | 29.55  39.51 |
| Protein | 43.59 | 85.81 | 35.22 | 38.75 |
| Ligands/ions | 55.72 | 72.21 | 43.51 | 51.47 |
| Waters | 38.90 | 55.85 | 43.59 | 35.69 |
| R.m.s. deviations |  |  |  |  |
| Bond lengths (Å) | 0.003 | 0.003 | 0.006 | 0.003 |
| Bond angles (°)  Ramachandran      Favored (%)      Allowed (%)      Outliers (%)  Rotamer outliers (%)  Clashscore | 0.59    99.00  1.00  0  0  4.15 | 0.66    97.85  2.15  0  0.18  6.57 | 0.77    99.03  0.97  0  0  4.07 | 0.56    97.84  2.16  0  0.55  3.60 |
