# Supplementary material for: Structural and Mechanistic Bases of Viral Resistance to HIV-1 Capsid Inhibitor Lenacapavir
Source: mBio. 2022 Oct 3;13(5):e01804-22. doi: 10.1128/mbio.01804-22 (PMC9600929; doi:10.1128/mbio.01804-22)

Synthesis of Subunit A

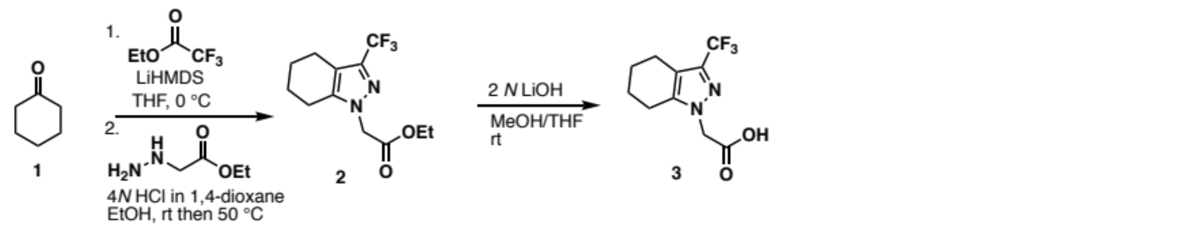

Synthesis of Subunit B

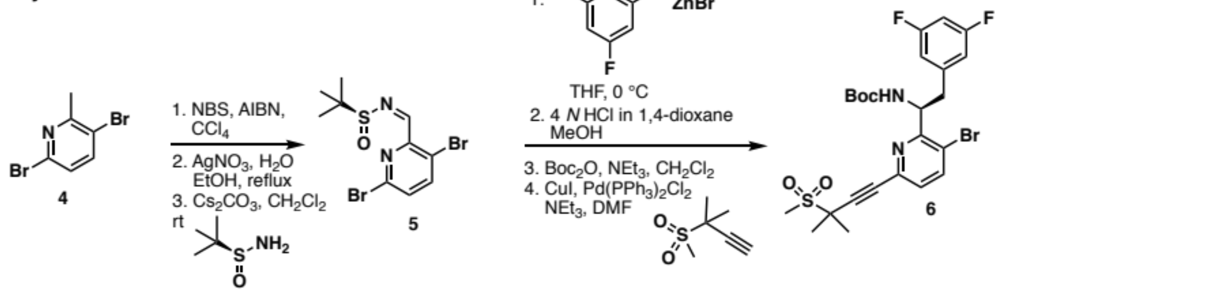

Synthesis of Subunit C

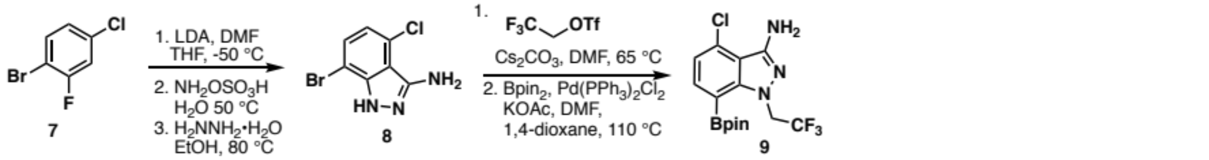

Synthesis of Compound 12

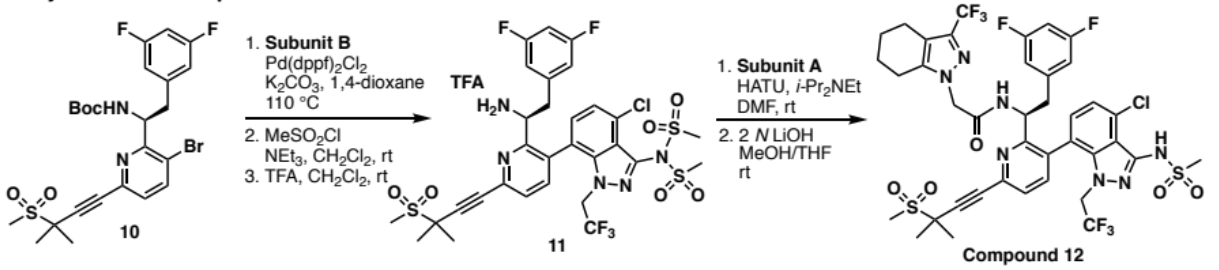

Supplement: Supplementary file 4 [file mbio.01804-22-s0005.docx]
